# A plant cell wall-associated kinase encoding gene is dramatically downregulated during nematode infection of potato

**DOI:** 10.1080/15592324.2021.2004026

**Published:** 2021-12-29

**Authors:** Shiyan Chen, Lili Cui, Xiaohong Wang

**Affiliations:** a School of Integrative Plant Science, Cornell University, Ithaca, NY, USA; b Rice Research Institute, Fujian Academy of Agricultural Sciences, Fuzhou, Fujian, China; c Robert W. Holley Center for Agriculture and Health, US Department of Agriculture, Agricultural Research Service, Ithaca, NY, USA

**Keywords:** Wall-associated kinase (*WAK*) and WAK-like kinase (*WAKL*) genes, potato, plant-parasitic cyst nematode, *Globodera rostochiensis*, promoter-GUS line

## Abstract

Plant cell wall associated kinases (WAKs) and WAK-like kinases (WAKLs) have been increasingly recognized as important regulators of plant immunity against various plant pathogens. However, the role of the *WAK*/*WAKL* family in plant-nematode interactions remains to be determined. Here, we analyzed a *WAK*-encoding gene (*Soltu.DM.02G029720.1*) from potato (*Solanum tuberosum*). The *Soltu.DM.02G029720.1* encoded protein contains domains characteristic of WAK/WAKL proteins and shows the highest similarity to SlWAKL2 from tomato (*S. lycopersicum*). We thus named the gene as *StWAKL2*. Phylogenetic analysis of a wide range of plant WAKs/WAKLs further revealed close similarity of StWAKL2 to three WAK/WAKL proteins demonstrated to play a role in disease resistance. To gain insights into the potential regulation and function of *StWAKL2*, transgenic potato lines containing the *StWAKL2* promoter fused to the β-glucuronidase (GUS) reporter gene were generated and used to investigate *StWAKL2* expression during plant development and upon nematode infection. Histochemical analyses revealed that *StWAKL2* has specific expression patterns in potato leaf and root tissues. During nematode infection, GUS activity was mostly undetected at nematode infection sites over the course of nematode parasitism, although strong GUS activity was observed in root tissues adjacent to the infection region. Furthermore, mining of the transcriptomic data derived from cyst nematode infection of Arabidopsis roots identified a few *WAK*/*WAKL* genes, including a *StWAKL2* homologue, found to be significantly down-regulated in nematode-induced feeding sites. These results indicated that specific suppression of *WAK*/*WAKL* genes in nematode-induced feeding sites might be crucial for cyst nematodes to achieve successful infection of host plants. Further studies are needed to uncover the role of *WAK*/*WAKL* genes in plant defenses against nematode infection.

## Introduction

Plant cell wall-associated kinases (WAK) and WAK-like kinases (WAKL) are a unique group of receptor-like protein kinases (RLKs) which are involved in many functions in plants, including plant development and plant immunity against pathogen infection.^1–3^ This group of RLKs contains an extracellular domain with similarity to the vertebrate epidermal growth factor (EGF)-like domain, a transmembrane (TM) domain, and an intracellular serine/threonine protein kinase domain.^4^ The Arabidopsis (*Arabidopsis thaliana*) genome encodes five WAKs and twenty-two WALKs.^5^ Arabidopsis *WAK*/*WAKL* genes have distinct and yet overlapping expression patterns, some of which are required for cell elongation and plant development.^2^ Recently, genome-wide analysis and characterization of the *WAK/WAKL* gene family has been reported for many other plant species including tomato (*Solanum lycopersicum*) and cotton (*Gossypium hirsutum*).^6,7^

WAK/WAKL proteins have been increasingly recognized as important contributors to disease resistance against bacterial and fungal pathogens.^1,3,7–15^ The Arabidopsis AtWAK1 is the best characterized WAK protein. AtWAK1 was shown to bind cell-wall-derived oligogalacturonides (OGs) and trigger OG-mediated defense responses effective against bacterial and fungal pathogens.^8^ Transgenic plants overexpressing *AtWAK1* are more resistant to the necrotrophic pathogen *Botrytis cinerea*.^8^ *AtWAKL22*, another member of the Arabidopsis *WAK*/*WAKL* family, is identified to encode a novel type of disease-resistance protein that confers resistance to a broad spectrum of Fusarium races.^9^ Consistently, Arabidopsis (Col-0 ecotype) mutated in *AtWAKL22* was more susceptible to Fusarium infection.^9^ In rice, a few *OsWAK* genes are revealed to be important regulators in rice resistance against the blast fungus *Magnaporthe oryzae*. Studies have revealed that several *OsWAK* genes including *OsWAK14, OsWAK25, OsWAK90*, and *OsWAK91* were upregulated upon infection by the blast fungus.^10,11^ Rice lines mutated in *OsWAK14* or *OsWAK91* were all more susceptible to the blast fungus, whereas lines with *OsWAK91* overexpression showed increased disease resistance.^10^ *OsWAK91* was further shown to be involved in ROS production and defense gene expression during pathogen infection.^10^ In tomato, *SlWAK1* plays an important role in plant immunity against the bacterial pathogen *Pseudomonas syringae* pv. tomato.^3^ *WAK*/*WAKL* genes involved in disease resistance have also been identified in other plant species including maize and cotton.^7,14,15^

Little is known about the *WAK*/*WAKL* gene family in potato. Potato is an economically important crop, but its production is threatened by various plant pathogens including potato cyst nematodes (PCN; *Globodera rostochiensis* and *G. pallida*). Cyst nematodes are soil-borne root pathogens. These endoparasitic nematodes actively interfere with host defenses, primarily through the action of their stylet-secreted effector proteins, to ensure the establishment of feeding cells within roots from which they drain the needed nutrients that ultimately results in disease symptoms.^16,17^ Studies have revealed that some plant RLKs, such as CLAVATA1 and RECEPTOR-LIKE PROTEIN KINASE 2 (RPK2), are required for cyst nematode parasitism.^16,18^ Our initial search of potato *RLK*s led to the identification of the *Soltu.DM.02G029720.1* gene. Primary sequence analysis indicated that *Soltu.DM.02G029720.1* encodes a WAK/WAKL protein. Although the *WAK*/*WAKL* gene family has been studied in many plant species and under both biotic and abiotic conditions, knowledge on the function of potato *WAK*/*WAKL* genes as well as a role of *WAK*/*WAKL* genes in plant-nematode interactions are mostly lacking. In this study, we analyzed the *Soltu.DM.02G029720.1* gene from potato and investigated its expression under normal plant growth conditions and upon nematode infection through the utilization of promoter-GUS lines.

## Results and discussion

### *Sequence analysis of* Soltu.DM.02G029720.1

**Figure 1.**
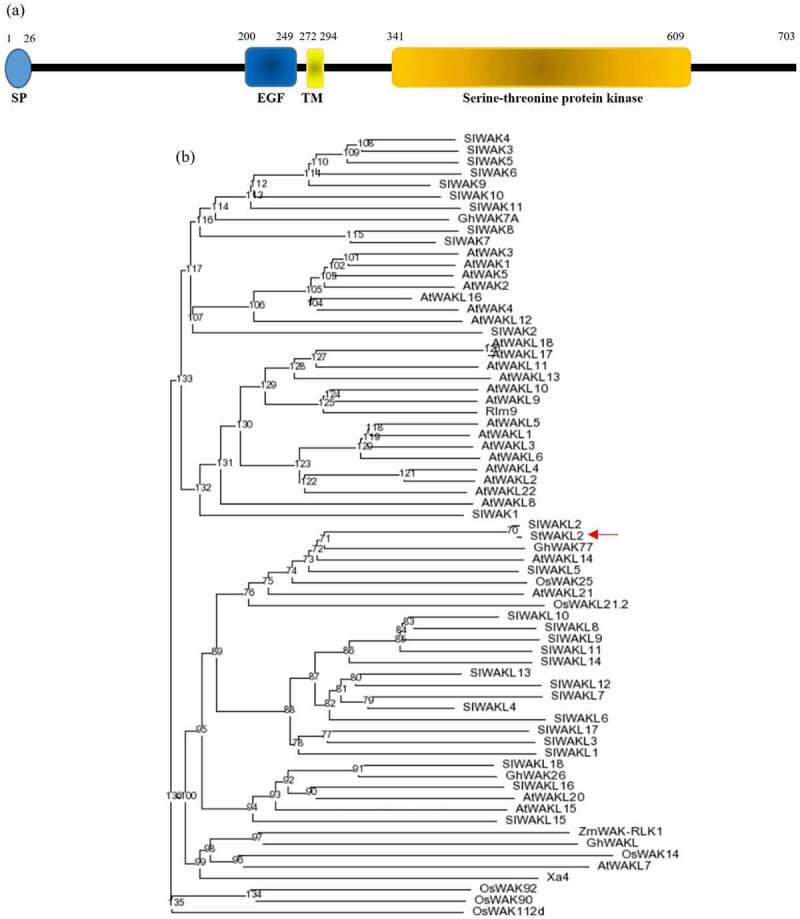
*StWAKL2* encoded protein and its relationship with other plant WAK/WAKL proteins. (a) Schematic representation of *StWAKL2* encoded protein. StWAKL2 was predicted to contain an N-terminal signal peptide (SP) and have domains characteristic of WAK/WAKL proteins including an extracellular epidermal growth factor (EGF)-like domain near the transmembrane (TM) region and a cytoplasmic serine/threonine protein kinase domain as indicated. (b) Phylogenetic relationship of StWAKL2 with other plant WAK/WAKL proteins. Multiple sequence alignment of StWAKL2 and plant WAK/WAKL2 protein sequences was obtained using the ClustalX program and the unrooted consensus tree from 1,000 bootstrap replicates was generated using the PHYLIP 3.61 program.^25^ Plant WAK/WAKL protein sequences included are those encoded by the *WAK*/*WAKL* gene family from Arabidopsis and tomato,^5−6^ as well as several other plant *WAK*/*WAKL* genes demonstrated to have a role in plant disease resistance.^7,10–15^

The *Soltu.DM.02G029720.1* gene is predicted to encode a protein of 703 amino acids which contains an N-terminal signal peptide and displays features of WAK/WAKL proteins, including an extracellular EGF-like domain near the transmembrane region and an intracellular serine/threonine protein kinase domain (Figure 1a). To better understand the relationship of the *Soltu.DM.02G029720.1* encoded protein with other plant WAK/WAKL proteins, we performed a phylogenetic analysis that includes all the Arabidopsis and tomato WAK/WAKL proteins and several other plant WAK/WAKL proteins shown to have a role in plant resistance against pathogen infection.^7,10,14^ The phylogenetic analysis revealed that the *Soltu.DM.02G029720.1* encoded protein is clustered with SlWAKL2 and SlWAKL5 from tomato, GhWAK77 from cotton, AtWAKL14 and AtWAKL21 from Arabidopsis, and OsWAK25 and OsWAKL21.2 from rice (Figure 1b). The Soltu.DM.02G029720.1 protein showed the closest similarity to tomato SlWAKL2 (98% similarity), followed by GhWAK77 (73% similarity), SlWAKL5 (72% similarity), and OsWAK25 (68% similarity) and OsWAKL21.2 (55% similarity) from cotton, tomato, and rice, respectively. The Soltu.DM.02G029720.1 protein has 58% and 63% similarity with AtWAKL14 and AtWAKL21, respectively, from Arabidopsis. As the *Soltu.DM.02G029720.1* encoded protein has the highest similarity with SlWAKL2, we thus name the gene as *StWAKL2*.

### Tissue-specific expression of StWAKL2 in potato

To gain insights into the potential regulation and function of *StWAKL2*, we initially analyzed the promoter region (2790 base pairs) upstream of the translation start site of *StWAKL2* to identify putative *cis*-acting elements using PlantCARE.^19^ Many phytohormone-responsive regulatory elements associated with auxin (AuxRE and TGA-element), methyl jasmonate (CGTCA-motif and TGACG-motif), abscisic acid (ABRE), and salicylic acid (SA) (TCA-element) were identified (Table 1). Stress-responsive regulatory elements, including TC-rich repeats and the WUN-motif involved in wounding and pathogen responsiveness, as well as elements associated with anaerobic induction (ARE) and drought inducibility (MBS) were also identified. Moreover, several light-responsive elements (I-box, G-box, TCT-motif, and AE-box) were found in *StWAKL2* promoter (Table 1). These results indicated that *StWAKL2* might have an important role in responses to hormones and stress signals.

**Table 1.** Putative *cis*-acting regulatory elements present in the *StWAKL2* promoter

Type of responsiveness	Number of *cis*-acting elements
*Auxin responsiveness*	
AuxRE	1
TGA-element	1
*Methyl jasmonic acid (MeJA) responsiveness*	
CGTCA-motif	1
TGACG-motif	1
*Abscisic acid (ABA) responsiveness*	
ABRE	1
*Salicylic acid (SA) responsiveness*	
TCA-element	1
*Defense and stress reaponsiveness*	
TC-rich repeats	1
*Wound responsiveness*	
WUN-motif	1
*Anaerobic induction and drought inducibility*	
ARE	2
MBS	1
*Light responsiveness*	
I-box	1
G-box	1
TCT-motif	2
AE-box	1

**Figure 2.**
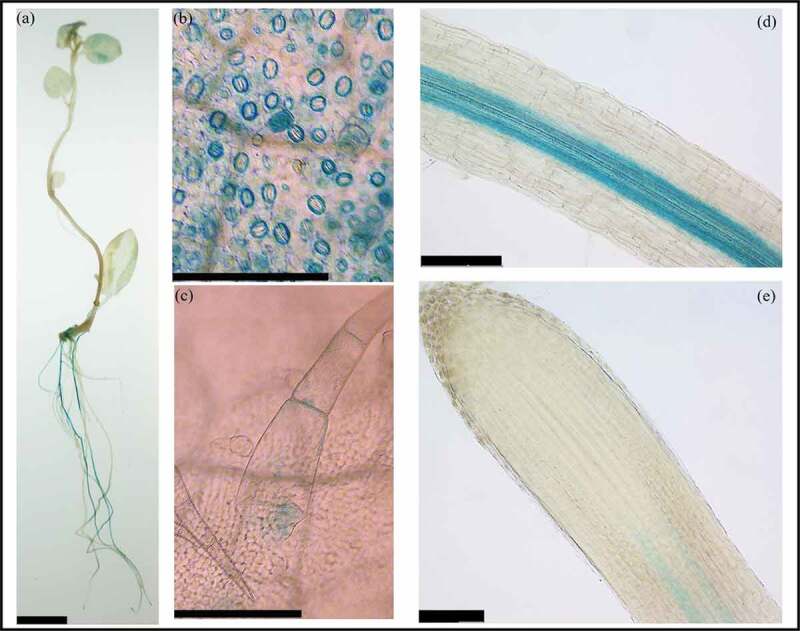
Tissue-specific expression of *StWAKL2* in potato. Transgenic potato lines expressing *StWAKL2pro-GUS* was generated and used to determine *StWAKL2* expression in potato tissues. Strong GUS staining was observed primarily in leaf and root tissues (a). A close examination of leaf and root tissues revealed GUS staining in leaf guard cells and trichomes (b-c) as well as throughout the root vasculature near the zone of maturation with no GUS staining in the root apical meristem (d-e). Scales bars = 1 cm in (a) and 200 µm in (b-e), respectively.

To further investigate the expression profile of *StWAKL2*, we cloned the 2790-bp promoter sequence of *StWAKL2* (Genbank accession number OK135347) into a binary vector to generate a *StWAKL2pro-GUS* construct, which was subsequently transformed into potato. Histochemical analysis of the obtained transgenic potato lines indicated that *StWAKL2* expression occurs in a tissue-specific manner. In tissue-cultured plantlets, GUS activity was primarily observed in leaves and roots (Figure 2a). A closer examination of the leaf tissue revealed specific GUS activity in stomatal guard cells and trichomes (Figure 2b-c). The rice *OsWAK11* gene was also found to be specifically expressed in leaf trichomes.^20^ Almost no GUS staining was observed in stems (Figure 2a). Within roots, GUS activity was observed throughout the vasculature starting near the zone of maturation, but no GUS activity was detected in the root apical meristem region (Figure 2d-e). In Arabidopsis, unlike *AtWAK* genes which are predominately expressed in green tissues (stems and leaves), *AtWAKL* genes are mainly expressed in roots.^2^ However, among the seven *AtWAKL* genes (*AtWAKL1-AtWAKL7*) that were characterized using promoter-GUS lines, none of them showed a similar expression pattern in roots as *StWAKL2. StWAKL2* might have an important function in leaf guard cells and trichomes as well as in root vasculature due to its specific expression in these tissues.

### StWAKL2 is dramatically suppressed during PCN infection of potato

**Figure 3.**
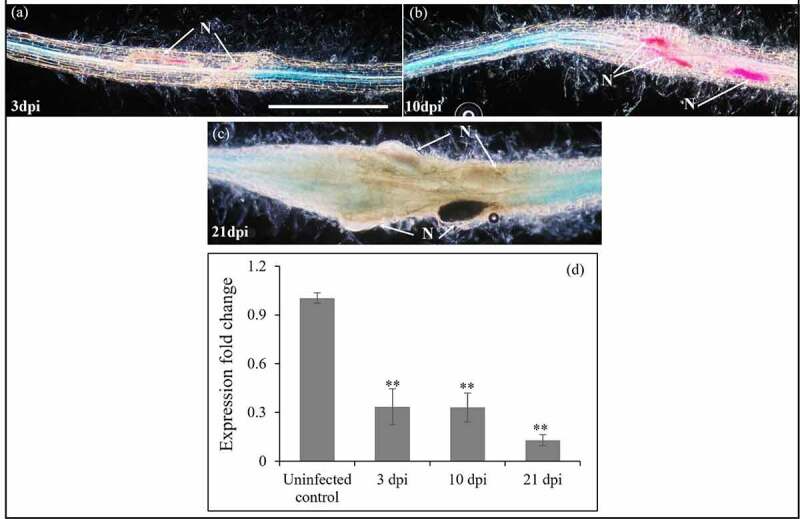
Downregulation of *StWAKL2* in response to nematode infection. (a) Roots of transgenic plantlets expressing *StWAKL2pro-GUS* were infected with potato cyst nematode (*Globodera rostochiensis*) and almost no GUS activity was detected at nematode infection sites that were associated with parasitic second-stage juveniles [at 3 days post inoculation (dpi) of nematodes] (a) and parasitic nematodes at later developmental stages (10 dpi and 21 dpi) (b-c). N = nematode; Scale bar = 1 mm. (d) Downregulation of *StWAKL2* during the course of nematode infection confirmed by RT-qPCR analysis.^22^ Values are means ± SD of three biological replicates (**P < .01, Student’s *t* test).

Prior to this study, knowledge on the involvement of the *WAK*/*WAKL* gene family in plant-nematode interactions was lacking. To evaluate a role of *StWAKL2* in nematode parasitism, transgenic *StWAKL2pro-GUS* potato lines were infected with potato cyst nematode *Globodera rostochiensis* and assayed for GUS activity over a time-course of nematode infection. Interestingly, almost no GUS staining was detected at nematode infection sites from the early to late stages of nematode infection, although strong GUS staining was detected in root tissues adjacent to the infection region (Figure 3a-c). We further used RT-qPCR to verify *StWAKL2* expression in response to nematode infection. As expected, the expression of *StWAKL2* was found to be dramatically reduced during nematode parasitism compared to that in uninfected potato roots (Figure 3d). The results likely correlate with the observation of the suppression of *StWAKL2* expression at nematode infection sites using *StWAKL2pro-GUS* lines. We also analyzed the transcriptomic data generated by Szakasits *et al*. (2009) on the study of cyst nematode infection of Arabidopsis roots.^21^ Among the twenty-one *WAK*/*WAKL* genes found to be expressed in Arabidopsis roots, five of them were revealed to be regulated in response to nematode infection. Interestingly, all the five *WAK*/*WAKL* genes including *AtWAKL14*, a *StWAKL2* homologue, showed significant down-regulation in nematode-induced feeding cells when compared to their expression in the normal root tissue (Table S1).^21^ Together, these results indicated that specific suppression of *WAK*/*WAKL* genes in nematode-induced feeding sites might be crucial for cyst nematode to achieve successful infection of host plants. Our phylogenetic analysis revealed that in addition to the four *WAKL* genes from Arabidopsis and tomato, *StWAKL2* has close similarity with *GhWAK77* from cotton and *OsWAK25* and *OsWAKL21.2* from rice (Figure 1b). All the three *WAK* genes have been implicated in plant immunity against bacterial and fungal pathogens. The cotton *GhWAK77* gene was significantly and constantly upregulated during infection by the soil borne fungus *Verticillium dahliae*.^7^ Silencing *GhWAK77* compromised cotton resistance to *V. dahliae*. It was suggested that *GhWAK77* might function in SA- and JA (jasmonate acid)-mediated signaling pathways to regulate cotton resistance against *V. dahliae*.^7^ Both *OsWAK25* and *OsWAKL21.2* were upregulated during pathogen infection.^11,13^ Furthermore, overexpression of *OsWAK25* resulted in elevated expression of several defense-related genes and increased plant resistance to the bacterial pathogen *Xanthomonas oryzae* and the blast fungus *Magnaporthe oryzae*.^11^ Both StWAKL2 and AtWAKL14 are grouped with GhWAK77, OsWAK25, and OsWAKL21.2 based on the phylogenetic analysis (Figure 1b). Due to this close relatedness, we hypothesize that StWAKL2 and AtWAKL14 may have a critical role in plant defenses against nematode infection. It would be interesting to determine if overexpression of *StWAKL2* or *AtWAKL14* would render host plants more resistant to cyst nematode infection.

In summary, we have analyzed a specific *StWAKL2* gene from potato through sequence comparison with a wide range of plant *WAK*/*WAKL* genes and used *StWAKL2pro-GUS* lines to reveal the tissue-specific expression and the negative regulation of *StWAKL2* during cyst nematode infection of potato. This study indicated for the first time that active regulation of specific members of the *WAK*/*WAKL* gene family might be critical for nematode parasitism of host plants.

## Materials and methods

### Nematode culture and plant materials

The potato cyst nematode *Globodera rostochiensis* was propagated on potato *(Solanum tuberosum)* and nematode infection assays on potato plantlets were conducted as previously described.^22,23^ Potato cv. Désirée was used for generating transgenic plants.^24^

### Sequence analysis of Soltu.DM.02G029720.1 encoded protein (named StWAKL2)

The SMART (simple modular architecture research tool) database (http://smart.embl-heidelberg.de/) and NCBI-CDD (http://www.ncbi.nlm.nih.gov/Structure/cdd/wrpsb.cgi) were used to identify domains in *Soltu.DM.02G029720.1* encoded protein sequence.

Phylogenetic analysis of plant WAK/WAKL proteins and characterization of the ***StWAKL2*** promoter region

Phylogenetic analysis of StWAKL2 with all the WAK/WAKL proteins from Arabidopsis and tomato and several other plant WAK/WAKL proteins demonstrated to have a role in plant defenses was performed as previously described.^25^ The PlantCARE database (http://bioinformatics.psb.ugent.be/webtools/plantcare/html/)^19^ was used to search for *cis*-acting elements in the *StWAKL2* promoter region (−2790 bp to −1 bp).

### Expression construct and potato transformation

A 2790-bp sequence upstream of the start codon of *StWAKL2* (Soltu.DM.02G029720.1) was amplified from potato (cv Désirée) genomic DNA by PCR using primers St2g23450_1-pro-SalI-F (5ʹ-CTCGTCGACCTGGTACAAAGCTTAGATCAACAC-3ʹ) and St2g23450_1-pro-BamH1-R (5ʹ-GAAGGATCCGGCTCGAAATTGAATCAAAG-3ʹ), and cloned into the binary vector pBI101.2,^26^ at the *Sal*I and *Bam*HI sites, to generate the *StWAKL2pro-GUS* construct. Transgenic potato lines expressing *StWAKL2p-GUS* were generated as described.^24,27^

### Histochemical GUS assay and nematode staining

Shoot tops cut from *in vitro*-grown transgenic potato plantlets were cultivated either in glass-tubes or in six-well plates containing the proper medium.^22,23^ Two weeks after growth, plantlets were either used for GUS staining to investigate the spatial expression of the *StWAKL2* gene or used for nematode infection.^22,23^ Roots at 3, 10, and 21 days post inoculation (dpi) of nematodes were collected and used for GUS staining followed by nematode staining. Non-infected plantlets and nematode-infected roots were infiltrated with GUS substrate buffer and incubated for 12–14 h at 37°C.^27^ Nematode staining was subsequently performed for infected roots collected at 3 dpi and 10 dpi.^27^ Stained roots were mounted on glass slides and visualized with a Nikon Eclipse TS100 inverted microscope.

### *StWAKL2* expression during nematode infection

mRNA from uninfected potato roots and *G. rostochiensis* infected root segments containing nematodes at 3, 10, and 21 days post inoculation (dpi) was extracted and used for RT-qPCR as previously described.^22^ Primers StWAKL-2_1308 F (5ʹ-AAAGCTCCACAGTGATGAATGG-3ʹ) and StWAKL-2_1526 R (5ʹ-CCCCTTTCTCTCTGTAGATGCT-3ʹ) were used to target *StWAKL2*, and primers StRPN7_F (5ʹ- GAGGGGAGGAATGCAGAT-3ʹ) and StRPN7_R (5ʹ-TCCATCTTCAAGCTGCTTACC-3ʹ) were used to target the potato 26S proteasome subunit gene *StRPN7* (Soltu.DM.07G005010.1) that served as an endogenous reference for data analysis. The RT-qPCR data were obtained from three independent experiments, with three technical replicates for each cDNA sample.

## Supplementary Material

Supplemental Material

## References

[1] Bacete L, Mélida H, Miedes E, Molina A. Plant cell wall-mediated immunity: cell wall changes trigger disease resistance responses. Plant J. 2018;93(4):614–6. doi:10.1111/tpj.13807. 29266460

[2] Kanneganti V, Gupta AK. Wall associated kinases from plants - an overview. Physiol Mol Biol Plants. 2008;14(1–2):109–118. doi:10.1007/s12298-008-0010-6. 23572878 PMC3550657

[3] Zhang N, Pombo MA, Rosli HG, Martin GB. Tomato wall-associated kinase SlWak1 depends on Fls2/Fls3 to promote apoplastic immune responses to *Pseudomonas syringae*. Plant Physiol. 2020;183(4):1869–1882. doi:10.1104/pp.20.00144. 32371523 PMC7401122

[4] Anderson CM, Wagner TA, Perret M, He ZH, He D, Kohorn BD. WAKs: cell wall-associated kinases linking the cytoplasm to the extracellular matrix. Plant Mol Biol. 2001;47(1–2):197–206. doi:10.1023/A:1010691701578. 11554472

[5] Verica JA, He ZH. The cell wall-associated kinase (*WAK*) and *WAK*-like kinase gene family. Plant Physiol. 2002;129(2):455–459. doi:10.1104/pp.011028. 12068092 PMC1540232

[6] Sun Z, Song Y, Chen D, Zang Y, Zhang Q, Yi Y, Qu G. Genome-wide identification, classification, characterization, and expression analysis of the wall-associated kinase family during fruit development and under wound stress in tomato (*Solanum lycopersicum* L.). Genes (Basel). 2020;11(10):1186. doi:10.3390/genes11101186. PMC7650724 33053790

[7] Yang J, Xie M, Wang X, Wang G, Zhang Y, Li Z, Ma Z. Identification of cell wall-associated kinases as important regulators involved in *Gossypium hirsutum* resistance to *Verticillium dahliae*. BMC Plant Biol. 2021;21(1):220. doi:10.1186/s12870-021-02992-w. 33992078 PMC8122570

[8] Brutus A, Sicilia F, Macone A, Cervone F, De Lorenzo G. A domain swap approach reveals a role of the plant wall-associated kinase 1 (WAK1) as a receptor of oligogalacturonides. Proc Natl Acad Sci U S A. 2010;107(20):9452–9457. doi:10.1073/pnas.1000675107. 20439716 PMC2889104

[9] Diener AC, Ausubel FM. RESISTANCE TO FUSARIUM OXYSPORUM 1, a dominant Arabidopsis disease-resistance gene, is not race specific. Genetics. 2005;171(1):305–321. doi:10.1534/genetics.105.042218. 15965251 PMC1456520

[10] Delteil A, Gobbato E, Cayrol B, Estevan J, Michel-Romiti C, Dievart A, Kroj T, Morel JB. Several wall-associated kinases participate positively and negatively in basal defense against rice blast fungus. BMC Plant Biol. 2016;16:17. doi:10.1186/s12870-016-0711-x. 26772971 PMC4715279

[11] Harkenrider M, Sharma R, De Vleesschauwer D, Tsao L, Zhang X, Chern M, Canlas P, Zuo S, Ronald PC. Overexpression of rice wall-associated kinase 25 (OsWAK25) alters resistance to bacterial and fungal pathogens. PLoS One. 2016;11(1):e0147310. doi:10.1371/journal.pone.0147310. 26795719 PMC4721673

[12] Hu K, Cao J, Zhang J, Xia F, Ke Y, Zhang H, Xie W, Liu H, Cui Y, Cao Y, *et al*. Improvement of multiple agronomic traits by a disease resistance gene via cell wall reinforcement. Nat Plants. 2017;3:17009. doi:10.1038/nplants.2017.9. 28211849

[13] Malukani KK, Ranjan A, Hota SJ, Patel HK, Sonti RV. Dual activities of receptor-like kinase OsWAKL21.2 induce immune responses. Plant Physiol. 2020;183(3):1345–1363. doi:10.1104/pp.19.01579. 32354878 PMC7333719

[14] Yang P, Praz C, Li B, Singla J, Robert CAM, Kessel B, Scheuermann D, Lüthi L, Ouzunova M, Erb M, *et al*. Fungal resistance mediated by maize wall-associated kinase ZmWAK-RLK1 correlates with reduced benzoxazinoid content. New Phytol. 2019;221(2):976–987. doi:10.1111/nph.15419. 30178602

[15] Wang P, Zhou L, Jamieson P, Zhang L, Zhao Z, Babilonia K, Shao W, Wu L, Mustafa R, Amin I, *et al*. The cotton wall-associated kinase GhWAK7A mediates responses to fungal wilt pathogens by complexing with the chitin sensory receptors. Plant Cell. 2020;32(12):3978–4001. doi:10.1105/tpc.19.00950. 33037150 PMC7721343

[16] Mitchum MG, Hussey RS, Baum TJ, Wang X, Elling AA, Wubben M, Davis EL. Nematode effector proteins: an emerging paradigm of parasitism. New Phytol. 2013;199(4):879–894. doi:10.1111/nph.12323. 23691972

[17] Hewezi T. Cellular signaling pathways and posttranslational modifications mediated by nematode effector proteins. Plant Physiol. 2015;169(2):1018–1026. doi:10.1104/pp.15.00923. 26315856 PMC4587465

[18] Guo X, Chronis D, De La Torre CM, Smeda J, Wang X, Mitchum MG. Enhanced resistance to soybean cyst nematode *Heterodera glycines* in transgenic soybean by silencing putative CLE receptors. Plant Biotechnol J. 2015;13(6):801–810. doi:10.1111/pbi.12313. 25581705

[19] Lescot M, Déhais P, Thijs G, Marchal K, Moreau Y, Van de Peer Y, Rouzé P, Rombauts S. PlantCARE, a database of plant *cis*-acting regulatory elements and a portal to tools for in silico analysis of promoter sequences. Nucleic Acids Res. 2002;30(1):325–327. doi:10.1093/nar/30.1.325. 11752327 PMC99092

[20] Hu W, Lv Y, Lei W, Li X, Chen Y, Zheng L, Xia Y, Shen Z. Cloning and characterization of the Oryza sativa wall-associated kinase gene *OsWAK11* and its transcriptional response to abiotic stresses. Plant Soil. 2014;384:335–346. doi:10.1007/s11104-014-2204-8.

[21] Szakasits D, Heinen P, Wieczorek K, Hofmann J, Wagner F, Kreil DP, Sykacek P, Grundler FM, Bohlmann H. The transcriptome of syncytia induced by the cyst nematode *Heterodera schachtii* in Arabidopsis roots. Plant J. 2009;57(5):771–784. doi:10.1111/j.1365-313X.2008.03727.x. 18980640 PMC2667683

[22] Chronis D, Chen S, Lu S, Hewezi T, Carpenter SC, Loria R, Baum TJ, Wang X. A ubiquitin carboxyl extension protein secreted from a plant-parasitic nematode *Globodera rostochiensis* is cleaved in planta to promote plant parasitism. Plant J. 2013;74(2):185–196. doi:10.1111/tpj.12125. 23346875

[23] Chronis D, Chen S, Lang P, Tran T, Thurston D, Wang X. *In vitro* nematode infection on potato plant. Bio-protocol. 2014;4(1):e1016. doi:10.21769/BioProtoc.1016.

[24] Chronis D, Chen S, Lang P, Tran T, Thurston D, Wang X. Potato transformation. Bio-protocol. 2014;4(1):e1017. doi:10.21769/BioProtoc.1017.

[25] Lu SW, Tian D, Borchardt-Wier HB, Wang X. Alternative splicing: a novel mechanism of regulation identified in the chorismate mutase gene of the potato cyst nematode *Globodera rostochiensis*. Mol Biochem Parasitol. 2008;162(1):1–15. doi:10.1016/j.molbiopara.2008.06.002. 18786575

[26] Jefferson RA, Kavanagh TA, Bevan MW. GUS fusions: beta-glucuronidase as a sensitive and versatile gene fusion marker in higher plants. EMBO J. 1987;6(13):3901–3907. doi:10.1002/j.1460-2075.1987.tb02730.x. 3327686 PMC553867

[27] Chen S, Lang P, Chronis D, Zhang S, De Jong WS, Mitchum MG, Wang X. *In planta* processing and glycosylation of a nematode CLAVATA3/ENDOSPERM SURROUNDING REGION-like effector and its interaction with a host CLAVATA2-like receptor to promote parasitism. Plant Physiol. 2015;167(1):262–272. doi:10.1104/pp.114.251637. 25416475 PMC4281011

